# P-834. NO-CAP: Navigating Overdiagnosis of Community-Acquired Pneumonia in Hospitalized Patients

**DOI:** 10.1093/ofid/ofaf695.1042

**Published:** 2026-01-11

**Authors:** Tiffany LaDow, William Ye, Hannah Angeles, Ellie Kang, Michal Stout, Tim Reynolds

**Affiliations:** Baylor Scott & White Medical Center - Temple, Georgetown, TX; Baylor Scott & White Medical Center - Temple, Georgetown, TX; University of Texas at Austin College of Pharmacy, Austin, Texas; Texas A&M College of Pharmacy, Temple, Texas; UT Tyler College of Pharmacy, Tyler, Texas; Baylor Scott & White Medical Center - Temple, Georgetown, TX

## Abstract

**Background:**

Inappropriate diagnosis of community-acquired pneumonia is common, especially in older patients and those with altered mentation, leading to inappropriate antibiotic use and adverse effects. The aim of this study is to compare the differences in antibiotic, steroid, and diuretic use from patients initially misdiagnosed with CAP in the emergency department (ED) to patients that were initially misdiagnosed but corrected by the time of discharge. Secondary aims are to describe the length of stay difference between the two groups.

**Methods:**

This was a single-center retrospective study of adults from July 1, 2023 to July 31, 2024 at a 660-bed teaching hospital in Central Texas. Patients were divided into two groups. The control group included patients who retained an inappropriate CAP diagnosis throughout their hospital stay (concordant group), and the comparator group included patients with an initial ED diagnosis of CAP not listed as a discharge diagnosis (discordant group). Inappropriate diagnosis was defined as patients with fewer than two signs or symptoms of CAP or negative chest imaging. Patients with sepsis, ICU admission, or another infectious disease requiring antibiotics were excluded. Chi-square and linear ANOVA tests were used to detect differences between the two groups. All statistical analysis was performed in R (R version 4.4.2).

**Results:**

Eighty-nine patients met inclusion criteria. There were 73 patients in the concordant group and 16 patients in the discordant group. Baseline characteristics were similar between groups. The average age was 70 years and 57.3% were male. Median duration of antibiotic (7 vs 8 days, p=0.8), diuretic (0 vs 1.5 days, p=0.4), and steroid (0 vs 0 days, p=0.4) therapy in the concordant vs discordant groups did not differ. Length of stay between the two groups (6.5 vs 7.3 days, p = 0.48) did not differ.

**Conclusion:**

Our study suggests that antibiotic use is prolonged in patients inappropriately diagnosed with CAP, whether that diagnosis persists until discharge or not. Overall, only a small percentage of patients (17.9%) initially inappropriately diagnosed had their diagnosis changed at discharge. Antibiotic stewardship opportunities remain alongside diagnostic stewardship in community-acquired pneumonia.

**Disclosures:**

All Authors: No reported disclosures

